# Origin of pyrite nodules at the top of the nantuo diamictites, Southern China

**DOI:** 10.1038/s41598-021-97022-y

**Published:** 2021-09-21

**Authors:** Changjie Liu, Ying Lin

**Affiliations:** 1 Department of Geology and Geophysics, Louisiana State University, Baton Rouge, LA USA; 2 Department of Geosciences, Texas Tech University, Lubbock, TX USA; 3 Department of Earth Sciences, University of California at Riverside, Riverside, CA USA

**Keywords:** Palaeoceanography, Palaeoclimate

## Abstract

Pyrite nodules up to 20 cm in diameter are found at the top of the Marinoan (~ 635 Ma) Nantuo glacial diamictite as well as in the cap dolostones and shale/siltstones in the lower Doushantuo Formation in eastern Guizhou, southern China. Field occurrences, petrography, and stable sulfur isotopic compositions of pyrite nodules were studied from a section at Taoying, eastern Guizhou, China. Pyrite δ^34^S values from different nodules varied from 7.3 to 60.5‰ at different stratigraphic levels. No stratigraphic trend existed for the δ^34^S, supporting the scenario of pyrite formation in sediments before the precipitation of the cap dolostone. Pyrite δ^34^S values were also homogeneous within individual nodules at a 0.3 to 1 cm sampling scale, but were more heterogeneous at a 2 mm sampling scale. Homogeneity was not expected from the particular model for pyrite nodule formation in a largely closed or semi-closed environment. Thus, differential cementation and compaction of the pyrite-bearing sediments may have produced the nodular shape of the pyrite deposit.

## Introduction

Sulfate (${\text{SO}}_{4}^{2 - }$) in modern seawater is 0.2% by weight, and is second only to chloride (Cl^-^) in concentration. Seawater sulfate concentration has varied over geological history. While periods of dramatic changes did occur, seawater sulfate concentration has generally increased over time. One of the extreme shifts in sulfate concentration was expected to have occurred at the aftermath of Marinoan global glaciations at ~ 635 Ma. Sulfate concentration is believed to be exceedingly low at the onset of deglaciation in the oceans. Peng et al.^1^ studied the occurrence of non-mass-dependently ^17^O depleted barite deposits in cap carbonates that drape the Nantuo diamictite, South China Block. They concluded that sulfate concentration in seawater was low or nearly absent during the deposition of the Doushantuo cap carbonates and the sulfate concentration in the oceans only rose after the deposition of cap dolostones, as evident from the first barite crystal fans being precipitated only at the top of reworked cap dolostones. Initially, shallow ocean sulfate had a significant riverine sulfate component, as supported by distinct negative Δ^17^O values (a measure of the δ^17^O deviation from what is expected from a mass-dependent relationship between the δ^17^O and δ^18^O) in these barite sulfates. The barium was supplied episodically to shallow oceans through the upwelling of deep Ba^2+^-rich water. This conclusion is echoed by the sequence of events occurring at the aftermath of Marinoan meltdown in the entire South China Block^2^.

In many shallow platform, shelf, and basinal facies of the South China Block, pyrite nodules of different sizes (up to 20 cm in diameter) occur at the top 0 to 2 m of the Nantuo diamictite, and occasionally within the cap dolostone of the basal Doushantuo Formation. Pyrite is usually precipitated through the reaction of dissolved sulfide produced by microbial sulfate reduction with Fe^2+^ derived from detrital iron-bearing minerals in anoxic marine sediments^3,4^. Pyrite precipitation can occur diagenetically in shallow sediments where both organic matter and sulfate are present in pore fluids, so that microbial sulfate reduction can produce sulfides (HS^–^ and H_2_S) to be precipitated as insoluble FeS. The initial FeS is later transformed to the more stable mineral pyrite (FeS_2_), the common sulfide minerals seen in the rock record^5,6^. Pyrite can also form in the water column. In a euxinic water column, dissolved sulfide reacts with free Fe^2+^ to form small FeS aggregates. Once the aggregates are larger than a critical size, they settle to bottom of the water column and are later transformed to pyrite^7,8^.

A scenario supporting the conclusion reached in Zhou et al.^2^ and Peng et al.^1^ would, therefore, predict that the basal Doushantuo pyrite nodules were formed in pore fluids after the deposition and disruption of the cap dolostones. By then, the ocean sulfate concentration had risen to a level that enough of it could diffuse into the pore fluids within the underlying sediments. Considering that the source of sulfate would be exclusively derived from the water column after the deposition of the cap dolostones and the Nantuo diamictite, this scenario predicts that the pyrite δ^34^S value would increase with depth, starting at the top of the cap dolostone.

Another possible scenario is that widespread pyrite formation in sediments could occur before the precipitation of the cap dolostone, either via direct precipitation of pyrite from a euxinic water column/in sediments or through in-sediment sulfate reduction. This scenario needs high enough seawater sulfate concentration or a euxinic water column before the deposition of cap dolostone. This scenario predicts that the many horizons of pyrite nodules at the top of the Nantuo diamictite may have large variability in their δ^34^S values and that the variation should have no relationship with depth.

**Figure 1:**
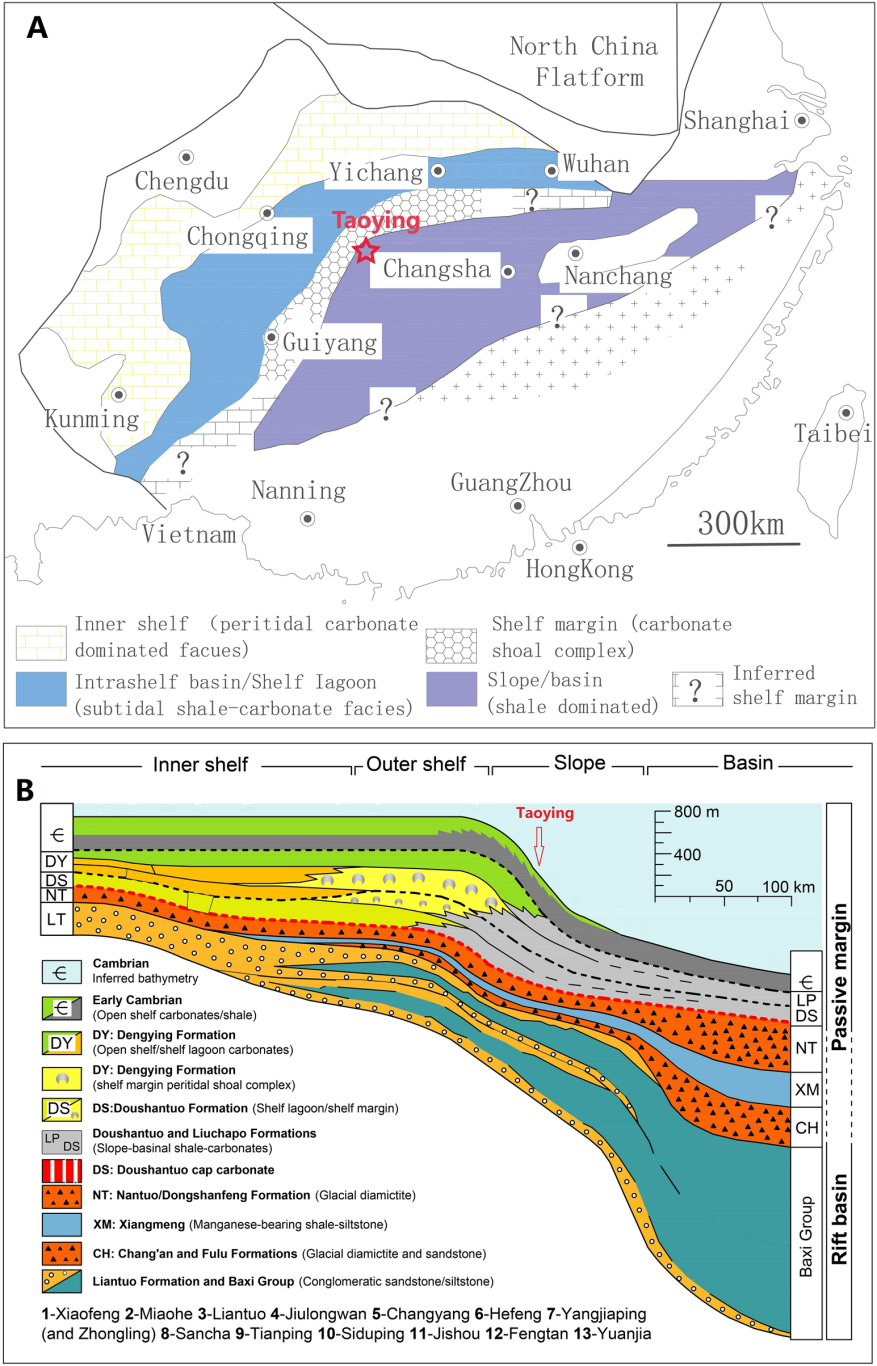
(**A**) a generalized paleogeographic reconstruction for the Yangze platform during Doushantuo deposition; (**B**) Shelf-to-basin transect from west to east in Guizhou and Hunan provinces^11^. The sample location is marked.

Lang et al.^9^ studied the pyrite concretions in the topmost Nantuo Formation in South China. Their results on sedimentary faces and sulfur isotope compositions of pyrite concretions indicated that pyrite precipitate in the sediments with H_2_S diffusing from the euxinic seawater. Lang et al.^9^ excluded the first scenario by the petrology of pyrite concretions (pyrite crystals are tightly packed with clasts or cemented in a siliciclastic matrix, and diamictite also contains disseminated pyrite) and the possibility of direct precipitation of pyrite from a euxinic seawater by pyrite morphology (Nantuo pyrite is euhedral instead of framboidal). This study evaluates these scenarios to explain the occurrence of the nodules in the South China Block. Although pyrite nodules have been observed in many facies in the Marinoan South China, we focused my study on samples from a well-exposed field section in Taoying, Tongling, eastern Guizhou (109°1′4.9"E, 27°50′1.4"N; Fig. 1). In summary, we examined the field occurrences, petrographic features, and stable sulfur isotope compositions (the δ^34^S) of pyrite nodules, together with a few pyrite lenses and beddings in the overlying Doushantuo shale and siltstones for comparison.

## Materials and methods

### Field occurrence of pyrite nodules

The Doushantuo Formation in the South China Block directly overlies the Nantuo glacial diamictite and consists of as much as 250 m of carbonates, siltstones, and shale^10–13^. In a well-exposed field section in Taoying, eastern Guizhou (Fig. 1), an about 1.4 m light-grey cap dolostones directly overlies a dark-grey Nantuo glacial diamictite. The cap dolostones are overlain by about 1.5 m of thinly-bedded dolostones followed by shales and siltstones full of pyrite lenses and beddings of the middle Doushantuo Formation (Fig. S1). Paleogeographically, Taoying is located on the slope between the platform and ocean basin (Fig. 1) Pyrite nodules of different sizes, ranging from invisible to the naked eye to ~ 20 cm in diameters, occur at the top 0–50 cm of the Nantuo diamictite, and occasionally within the cap dolostone of the basal Doushantuo Formation at Taoying (Fig. S1). Multiple nodules are also seen in the same horizons at the top of the diamictite.

### Petrography

**Figure 2:**
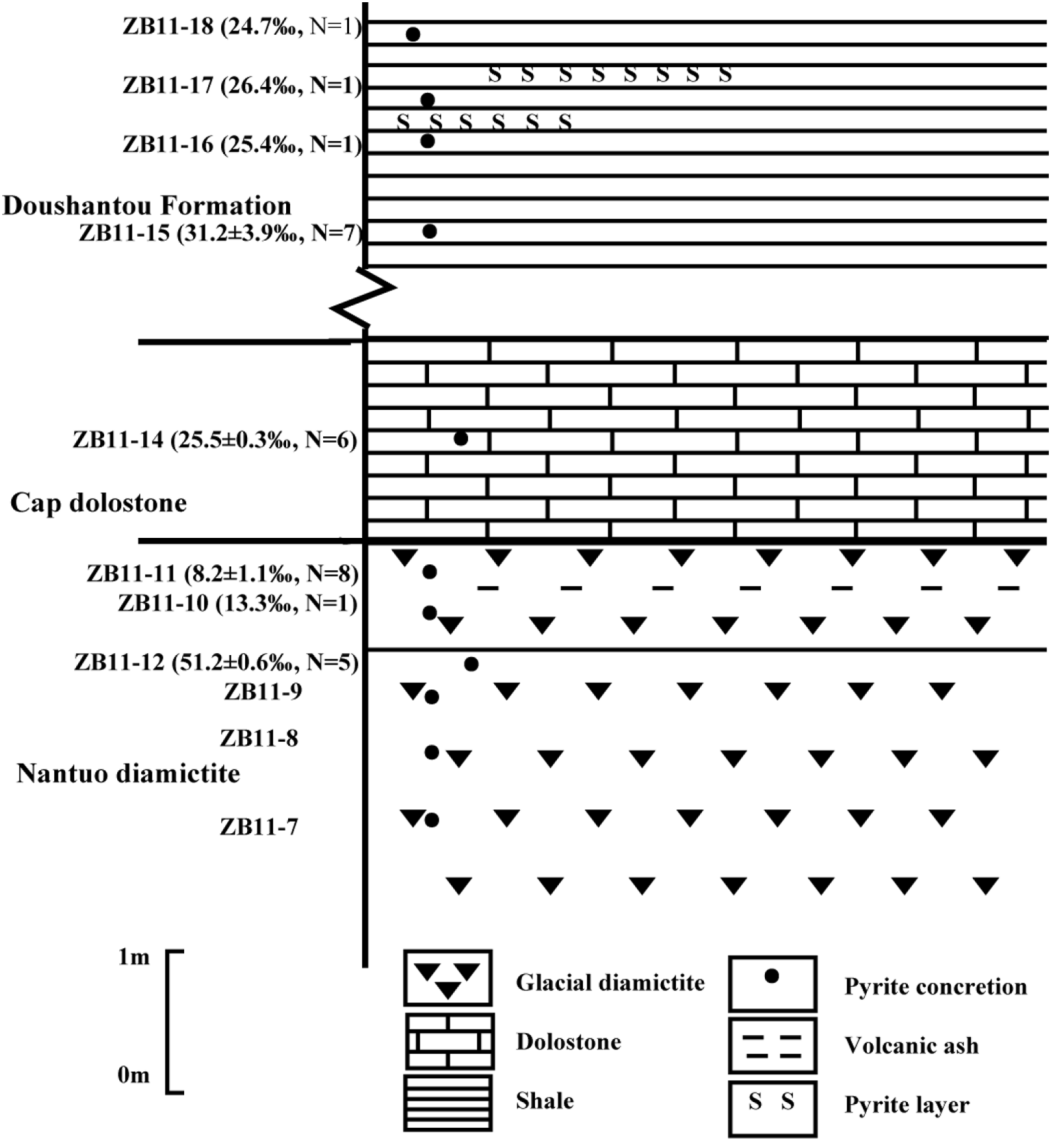
Stratigraphic column showing the distributions and δ^34^S (‰ VCDT) values of the analyzed pyrite nodules at the top of Nantuo glacial diamictite, the overlying cap dolostones of the Doushantuo Formation in Taoying, Tongling, eastern Guizhou. The values in the brackets are average of δ^34^S and the number of samples analyzed. ZB11-7, -8, -9 are bulk diamictite samples, and other are pyrite nodules or lenses.

Samples ZB11-7, ZB11-8, and ZB11-9 were bulk diamictite samples. Samples ZB11-10, ZB11-11, and ZB11-12 were pyrite nodules in the diamictite. ZB11-14 was a pyrite nodule in the cap dolostones (Fig. S1). ZB11-15a, b, c, d, e were five individual pyrite nodules in the shale at ~ 19 m above the top of the diamictite. Going further upward in stratigraphic level (22 m above the cap dolostones), pyrite nodules, ZB11-16, ZB11-17, and Zb11-18 were collected. The distribution of pyrite nodules collected at the top of Nantuo glacial diamictite, cap dolostones, and overlying shale are shown in Fig. 2. Thin sections were made from the bulk diamictite samples (ZB11-7, ZB11-8, ZB11-9), and pyrite nodules (ZB11-11 and ZB11-12), and a polished slab was made for the nodule ZB11-14. Sample ZB11-10 was too small to make a thin section. Photomicrographs were taken for thin sections and polished slabs using reflected light microscopes and a digital camera (Fig. S2).

The X-ray Diffraction (XRD) analysis was conducted on eight sample powders, including both bulk diamictite and pyrite nodules, using a Bruker/Siemens D5000 X-ray Diffractometer. The samples were run from 2° to 70° at a rate of 0.02° every 2 s with a setting of 40 kV and 30 mA. The diffraction pattern data were analyzed using Jade 9.3.3 software to confirm mineral identification from Material Data Incorporated. The quantitative analysis was obtained from XRDPHil program.

### Stable isotopic analysis

Field and initial petrographic observations revealed that fine-grained pyrite crystals or aggregates were common at the top of the diamictite. To examine the spatial heterogeneity of pyrite sulfur isotope compositions at different stratigraphic levels, among different nodules, or within the same nodule, both the bulk diamictite and pyrite nodules were collected. For picking a pyrite sample for δ^34^S analysis, only ~ 30 μg of pure pyrite was needed. To sample pyrite nodules, a piece of a nodule was broken into many smaller pieces, and then each was ground into fine powders. Approximately 30 μg was used per piece. Depending on the size of the overall nodule, samples were taken at sizes between 0.3 and 1.0 cm. ZB11-11, -12, -14, and -15a were sampled for δ^34^S analysis on the same nodule, with spatial difference. All other nodules, lenses, or beds only had one δ^34^S measurement for each. The stratigraphic positions for these samples are shown in Fig. 2. In total, δ^34^S data of 29 samples from 12 pyrite nodules or lenses were obtained at centimeter sampling resolution.

**Figure 3:**
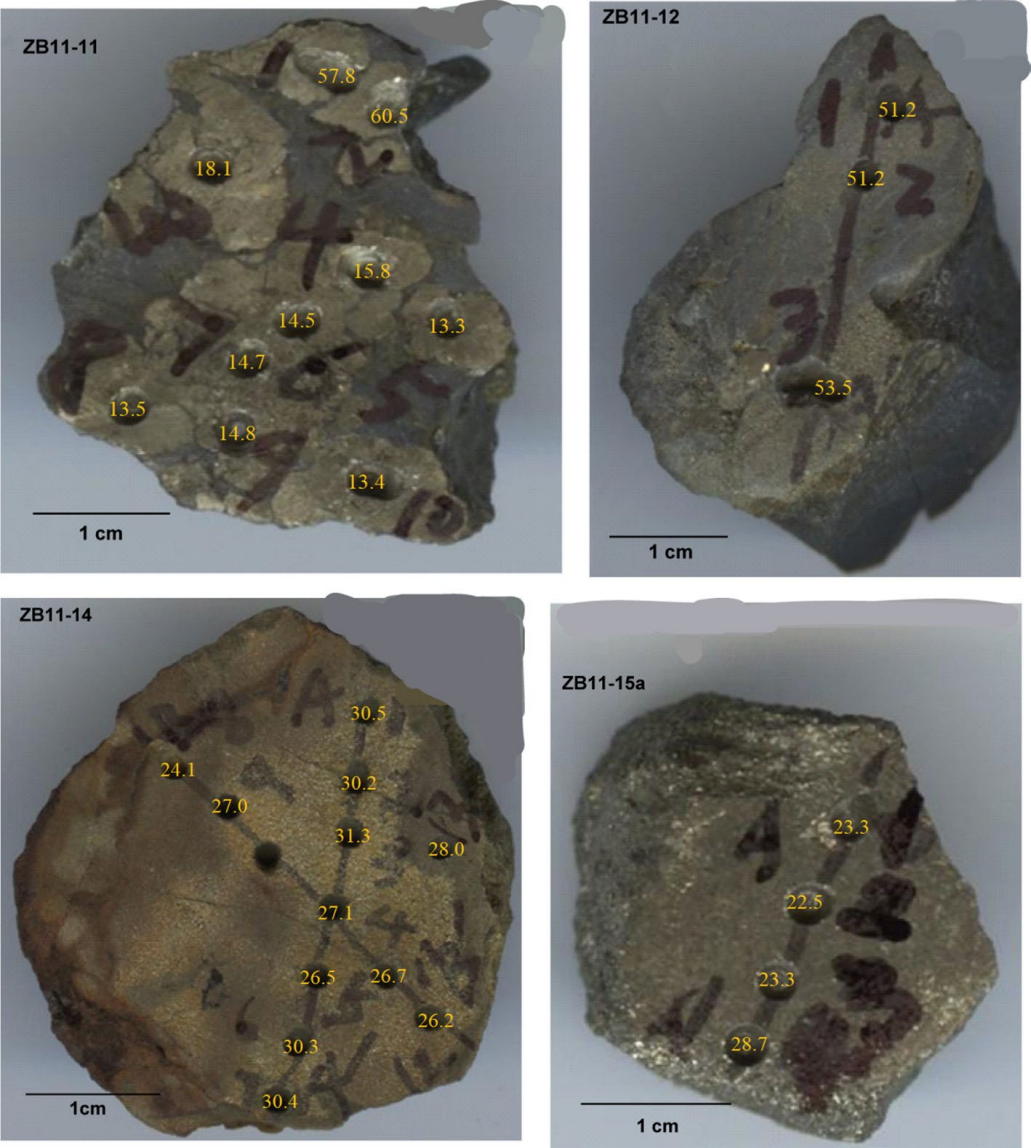
Millimeter-resolution sampling of pyrite nodules with their δ^34^S values displayed.

To further examine the potential spatial heterogeneity of the pyrite δ^34^S values within and between nodules, pyrite nodules ZB11-11, ZB11-12, ZB11-14, and ZB11-15a were sampled with smaller distances between each sample, at approximately 2 mm apart, from polished pieces of the pyrite nodules (Fig. 3). Powder was drilled out of each sample, yielding 30 additional data points.

For δ^34^S measurements of bulk diamictite samples, samples ZB11-7, ZB11-8, and ZB11-9 were taken before visual inspection of their corresponding thin-sections. A 10% wt FeS_2_ was assumed, and about 300 μg bulk materials were weighed out for sulfur isotope measurement of the sulfides in the sample.

Petrographic preparation, microscopic observation, sample milling and weighing were carried out at Louisiana State University and the millimeter sampling and δ^34^S measurement of sulfide was conducted at University of Maryland, where FeS_2_ was converted to SO_2_ using an Elemental Analyzer (EA) at 1050 °C, and analyzed on a Micromass Isoprime in a continuous-flow mode. The standard deviation associated with δ^34^S measurement was ± 0.2‰. All δ^34^S values are reported with respect to VCDT.

## Results

Microscopic observation revealed that disseminated pyrite grains were ubiquitous in the Nantuo diamictite. Within pyrite nodules, the individual pyrite grains occurred as aggregates. Although pyrite grain distribution density varied between nodules, the distribution was homogeneous at a millimeter scale. However, uneven distribution of pyrite and surrounding silicate matrix was observed on the scale of tens of micrometers (not considering some of the vein fillings). There was a general increase in pyrite abundance towards the top of the diamictite from sample ZB11-7 to sample ZB11-11.

XRD analysis of four bulk diamictite samples and four pyrite nodule samples confirmed that the iron sulfide mineral in the nodules was pyrite (Table 1). Other than pyrite, the significant minerals in the nodules were quartz and clay. In bulk diamictite, pyrite accounted for less than 2% of the weight, but in nodules the percentage of pyrite was at least more than 50%.

**Table 1:** Mineral composition and estimated weight percentage (± 10%) in bulk diamictite and pyrite nodule samples.

Sample name	Sample type	Clay	Quartz	Plagioclase	Dolomite	Pyrite	k-feldspar	Gypsum
ZB11-7	Diamictite	49	47	2	2			
ZB11-8	Diamictite	42	52	1	2			
ZB11-9	Diamictite	41	53	1	2	2		
ZB11-10	Pyrite nodule	48	34			16	2	
ZB11-12	Pyrite nodule		19			80		1
ZB11-11	Pyrite nodule	22	28			50		1
ZB11-14	Pyrite nodule					100		
ZB11-15a	Pyrite nodule		1			99		

The δ^34^S values for pyrite nodules sampled at a centimeter scale are shown in Table 2. The δ^34^S values varied from one pyrite nodule to another (from 7.3 to 51.6‰). However, δ^34^S values were homogeneous within the same pyrite nodule at the 0.3–1.0 cm sampling resolution (Table 2). For the three pyrite nodule samples in the diamictite, δ^34^S values ranged from 7.3 to 9.3‰ (average 8.2‰, N = 8) for ZB11-11, 13.3‰ (only one measurement) for ZB11-10, and ranged from 50.6 to 51.6‰ (average 51.2‰, N = 5) for sample ZB11-12. The δ^34^S valued for pyrite in the cap dolostones, ZB11-14, ranged from 25.2 to 25.8‰, with an average value of 25.5‰ (N = 5). The δ^34^S values for the 5 individual pyrite lenses collected in the shale overlying the cap dolostones, ZB11-15a, b, c, d and e, ranged from 27.2 to 31.7‰. Note that three samples were collected from pyrite lens ZB11-15a with a δ^34^S value range of 27.3–32.0‰, indicating higher heterogeneity than the nodules from the top of the diamictite and from within the cap dolostones. For the three pyrite lenses in the overlying shale, ZB11-16, ZB11-17, and ZB11-18, the δ^34^S values were 25.4‰, 26.4‰ and 24.7‰, respectively (Table 2).

**Table 2:** Sulfur isotope composition of pyrite nodules in Taoying, Guizhou Province, southern China; sampled in 0.3 to 1.0 cm spatial resolution.

Sample name	Sample number	Δ^34^S (‰ VCDT)	Sample name	Sample number	Δ^34^S (‰ VCDT)
ZB11-10	ZB11-10	13.3	ZB11-14	ZB11-14-1	25.8
ZB11-11	ZB11-11-1	8.0		ZB11-14-2	25.3
	ZB11-11-3	7.5		ZB11-14-3	25.2
	ZB11-11-4	8.2		ZB11-14-4	25.7
	ZB11-11-5	7.3		ZB11-14-5	25.6
	ZB11-11-6	9.3		ZB11-14-6	25.4
	ZB11-11-7	8.7	ZB11-15a	ZB11-15a-1	32.0
	ZB11-11-8	7.4		ZB11-15a-2	30.7
	ZB11-11-9	9.0		ZB11-15a-3	27.3
ZB11-12	ZB11-12-1	51.6	ZB11-15b	ZB11-15b	34.2
	ZB11-12-2	51.3	ZB11-15c	ZB11-15c	30.9
	ZB11-12-3	51.1	ZB11-15d	ZB11-15d	31.6
	ZB11-12-4	50.6	ZB11-15e	ZB11-15e	31.7
	ZB11-12-5	51.4	ZB11-16	ZB11-16	25.4
			ZB11-17	ZB11-17	26.4
			ZB11-18	ZB11-18	24.7

The results of pyrite sulfur isotope analysis from the 2 mm sampling scale are shown in Fig. 3 and Table 3. The δ^34^S values were more heterogeneous than those obtained with the larger sampling scale. Sample ZB11-12 values were more or less the same (~ 51.2‰) at both sampling resolutions. The δ^34^S values from sample ZB11-14 ranged from 24.1 to 31.3‰ on the millimeter scale, which was a larger range than at the larger sampling scale (25.2 to 25.8‰). The δ^34^S values for sample ZB11-15a was ~ 23‰ at the fine sampling scale, which was different than at the wider interval (~ 30‰). Sample ZB11-11 was an interesting case. There appeared to be multiple aggregates in the same individual nodule that had very different δ^34^S values, 14‰, 57.8‰, and 60.5‰, as compared to the centimeter-resolution value of ~ 8‰.

**Table 3:** Sulfur isotope composition of pyrite nodules in Taoying, Guizhou Province, southern China; sampled in 2 mm spatial resolution.

Sample name	Sample number	Δ^34^S (‰ VCDT)	Sample name	Sample number	δ^34^S (‰ VCDT)
ZB11-11	ZB11-11-1	57.8	ZB11-14	ZB11-14-1	30.5
	ZB11-11-2	60.5		ZB11-14-2	30.2
	ZB11-11-3	18.1		ZB11-14-3	31.3
	ZB11-11-4	15.8		ZB11-14-4	27.1
	ZB11-11-5	13.3		ZB11-14-5	26.5
	ZB11-11-6	14.5		ZB11-14-6	30.3
	ZB11-11-7	14.7		ZB11-14-7	30.4
	ZB11-11-8	13.5		ZB11-14-8	24.1
	ZB11-11-9	14.8		ZB11-14-9	27.0
	ZB11-11-10	13.4		ZB11-14-11	26.7
ZB11-12	ZB11-12-1	51.2		ZB11-14-12	26.2
	ZB11-12-2	51.2		ZB11-14-13	28.0
	ZB11-12-3	53.5	ZB11-15a	ZB11-15a-1	23.3
				ZB11-15a-2	22.5
				ZB11-15a-3	23.3
				ZB11-15a-4	28.7

## Discussion

During microbial sulfate reduction, sulfate is reduced to sulfide with sulfide exhibiting much lower δ^34^S values than the sulfate that it was derived from. Lab experiments showed that the sulfur isotope fractionation factor between sulfide and sulfate during dissimilatory microbial sulfate reduction varies between − 66‰ ~  − 0‰, depending on factors such as sulfate concentration, sulfate reduction rate, and temperature^14–19^. In natural environments, the δ^34^S difference between sulfide and sulfate could be as large as − 76‰^20–23^, due to the reservoir effect, sulfur oxidative recycling, and/or microbial sulfur disproportionation^7–10^. The reservoir effect often dominates the pyrite δ^34^S distribution in sediments. For a closed reservoir, according to the Rayleigh Model, the δ^34^S value of produced sulfide will increase due to an increasing δ^34^S value for the remaining sulfate, whether the fractionation factor remains the same or decreases with decreasing sulfate concentration.

Our first scenario proposes that there was little sulfate in seawater during the deposition of the diamictite and cap dolostones. Thus, even though there was plenty of organic matter being buried in the sediments, sulfate reduction did not occur. Later, a basin-wide transgression flooded the cap dolostones and sulfate concentration in seawater rose to significant levels since weathered sulfides with continental sulfate washed into the oceans^1,2^. At this time, organic matter in the cap dolostones and in the diamictite began to be oxidized, such as through microbial sulfate reduction. As sulfate diffused downward from the ocean water column, and was consumed by sulfate reducing microbes, the δ^34^S values of the sulfate in the upper horizon of the cap dolostone and diamictite would be less positive than in the deeper horizons due to preferential reduction of ^34^S-depleted sulfate that would potentially form pyrite. Because little to no sulfate reduction occurred during the deposition of the diamictite, all sulfate came from the top. This scenario predicts that the pyrite δ^34^S value would increase with increasing depth in the diamictite. Due to the widespread occurrence of fractures in the cap dolostones, the sulfate reservoir in cap dolostones would be much closer to an open system than that in the more compacted diamictite. Thus, such a depth-δ^34^S trend may not be expected in the cap dolostones.

However, if seawater sulfate during the waning stage of diamictite deposition was high enough so that concurrent sulfate reduction could occur, then pyrite could be syngenetic (formed in sediments via direct precipitation in a euxinic water column) or diagenetic (formed via in-sediment sulfate reduction or sulfide diffusion). In this second scenario, pyrite formation in the diamictite would have occurred continuously at different times and at different depths. The highly variable sedimentation rate^23^, sulfate concentration, organic content, sediment type, and microbial activity in this scenario would result in highly variable pyrite δ^34^S values from horizon to horizon with no correlation with stratigraphic depth.

Sedimentary pyrite can form in the water column or in sediments. Pyrite formation requires active iron and sulfide present, but because sulfide is usually produced by microbial sulfate reduction in an anoxic environment, a biogenic origin for pyrite formation requires sulfate-reducing microbes that use sulfate as electron acceptor and organic matter as an electron donor (Eq. 1).

The source for reduced iron for pyrite can be from ferric iron-bearing minerals in detrital sediments, such as ferrihydrite, geoethite, hematite, and lepidocrocite^4^. These minerals supply Fe (II) when they are reduced in anoxic environments either abiotically or microbially.

Dissolved sulfide reacts with Fe (II), and precipitates as FeS, mackinawite (tetragonal Fe_(1+x)_S, x≈0.05) or gregite (cubic Fe_3_S_4_). All three of these mineral phases are not thermodynamically stable and they transform to pyrite eventually (Eqs. 2 and 3).^3,24^ According to our XRD results, the transformation from the initial iron-sulfide forms to pyrite is complete because all of the samples contained pyrite.

1 $$2{\text{CH}}_{2} {\text{O }} + {\text{ SO}}_{4}^{2 - } \to {\text{H}}_{2} {\text{S }} + \, 2{\text{HCO}}_{3}^{2 - }$$

2 $${\text{Fe}}^{2 + } + {\text{ S}}^{2 - } \to {\text{FeS}}$$

3 $${\text{FeS}} + {\text{S}} \to {\text{FeS}}_{2} \left( {{\text{pyrite}}} \right)$$

The difference between the two proposed scenarios is the timing of pyrite formation, which is critical to understanding the ending of Marinoan global glaciations and the recovery of the biosphere at that time. The scenario that pyrite nodules were diagenetically formed after the precipitation of the cap dolostones predicts a gradual increase in pyrite δ^34^S values with increasing depth into the diamictite. The scenario that pyrite formed continuously at the waning stage of diamictite deposition predicts a highly variable pyrite δ^34^S value from horizon to horizon with no correlation with depth. Therefore, the vertical δ^34^S pattern for pyrite nodules collected from the diamictite can test which scenario is more likely in our geological settings. At the centimeter sampling resolution, pyrite δ^34^S values seem to increase with depth into the diamictite: average δ^34^S of ZB11-11 is 8.2‰, the δ^34^S of ZB11-10 is 13.3‰, and average δ^34^S of ZB11-12 is 51.2‰ (Fig. 2, Table 2). However, this trend is not supported in the millimeter sampling resolution—the δ^34^S of ZB11-11 ranges from 13.3 to 18.2‰ with two outliers (57.8‰ and 60.5‰) and the δ^34^S of ZB11-12 ranges from 51.2 to 53.5‰ (Fig. 3, Table 3). Thus, we conclude from these different datasets that pyrite δ^34^S values are highly variable from horizon to horizon with no correlation with depth (Fig. S4a), implying that seawater sulfate concentration may be still relatively low but already high enough to result in pyrite formation in sediments at the waning stage of diamictite deposition and before the precipitation of the cap dolostones, either via direct precipitation of pyrite or sulfide diffusion into sediments from a euxinic water column or via in-sediment sulfate reduction. Lang et al.^9^ discussed the formation mechanism of pyrite concretions at the same time using pyrite petrology and geochemical models based sulfur isotope composition of pyrite, and their results show that the water column is euxinic at the time and the pyrite formed though sulfide diffusing from the water column into sediments and reacting with iron ions in the sediments. Their conclusions are consistent with ours and further point out that the sulfide diffusion into sediments is the more likely pathway for pyrite formation.

Although the two different pyrite formation scenarios can be differentiated by the proposed stable sulfur isotope ratio analysis, the nodular form of the pyrite occurrence in the cap dolostones and the diamictite needs to be explained. Berner^25^ proposed a model for the formation of at least one type of pyrite concretions (Fig. S5). In his model, a small mass of organic matter was deposited in sediments of otherwise generally low organic content in a reducing micro-environment and with a high concentration of iron. When the sulfide ions diffuse radially out from the organic source during sulfate reduction, the ions would be trapped close to the organic source by reactive iron, e.g., Fe^2+^. The dissolved iron could then diffuse radially towards the organic center and precipitate at organic source boundaries through the precipitation of FeS. Continuous processes like this could result in the formation of an iron sulfide concretion surrounding and enclosing a body of organic matter^25^.

Berner’s pyrite concretion formation model would predict that within a single individual pyrite concretion, the δ^34^S would be heterogeneous, with the center of a concretion have a lower value and the outer ring having a higher value with respect to δ^34^S^25^. To check this model, the δ^34^S values in different parts of individual pyrite nodule were sampled and measured. If the predicted pattern was not observed, then an alternative pyrite nodule formation model was needed.

Pyrite nodules have been reported in shales from lake and marine sequences^26–29^. Raiswell^30^ sampled two pyritiferous carbonate nodules (30 cm and 70 cm in diameter) for sulfur isotope analysis. Five to six samples from the nodule center to the edge were collected from a slice of the nodules, and the δ^34^S values for pyrite increased ~ 8 and ~ 21‰, respectively, from the center to the edge for two nodules sampled. These data are consistent with a restricted sulfate reservoir being progressively depleted by microbial sulfate reduction.

Consequently, the only pyrite nodule formation model that has been proposed, by Berner^27^, predicts that δ^34^S values from pyrite will increase from center to edge in a pyrite concretion due to a reservoir effect. This model, however, cannot explain the spatially homogeneous δ^34^S values within the pyrite nodules in our study, at either one of the sampling intervals. Although millimeter sampling resolution revealed more heterogeneity, the δ^34^S values of pyrite were more or less within ± 2‰ of each other in one single aggregate. It is, therefore, apparent that Berner’s pyrite concretion model does not apply to the pyrite nodules at the top of the Nantuo diamictite in South China.

It is possible that the pyrite nodules at the top of the Nantuo diamictite were initially deposited as layers of disseminated pyrite grains or framboidal clusters. Due to differences in early cementation rates between pyrite layers and surrounding fine silicate muds, sedimentary compaction can turn the layered pyrite into nodular form of semi-linked and later totally independent pyrite nodules (Fig. S6). The nodule formation model by differential cementation and compaction of the sediments has been applied to explain carbonate concretion formation^10^. Such a pyrite nodule formation model can explain the δ^34^S homogeneity within a nodule.

## Conclusions

There are two scenarios to explain when and how the pyrite nodules in the Nantuo diamictite formed. One scenario is that these pyrite nodules formed diagenetically after the cap dolostone deposition when the seawater sulfate concentration became high enough to sufficiently diffuse into the diamictite. Another scenario is that the pyrite formed at the waning stage of diamictite deposition before the cap dolostone deposition when seawater sulfate concentration was sufficient to support microbial sulfate reduction. The difference between these two scenarios is in their predictions about the relationship between the δ^34^S of pyrite and depth. Our results show that pyrite δ^34^S values have no correlation with depth in the diamictite. Therefore, we conclude that the pyrite formed before the deposition of cap dolostones, and at that time the sulfate concentration may be relatively low but was high enough for microbial sulfate reduction. Pyrite could form in sediments via direct precipitation or sulfide diffusion from a euxinic water column or via in-sediment sulfate reduction. In any case, sulfate concentrations had to be sufficiently high, at least intermittently, in the oceans before the deposition of the cap dolostones. This conclusion has important implications in our understanding of the post-glacial world 635 million years ago. The globally distributed cap dolostones on top of the Marinoan diamictite has been concluded to be deposited immediately and continuously following the diamictite^31,32^. It becomes clear from this study that there was a time window when sulfur cycling is especially active before the cap dolostones deposition.

Berner’s pyrite nodule formation model does not apply to the pyrite nodule formation largely because of the observed pyrite δ^34^S homogeneity within a pyrite nodule. We propose that differential cementation and compaction of the pyrite-bearing sediments formed the rounded shapes of the pyrite nodules and can account for the δ^34^S homogeneity within an individual nodule.

## Supplementary Information

Supplementary Information.
