# Supplementary material for: Origin of pyrite nodules at the top of the nantuo diamictites, Southern China
Source: Sci Rep. 2021 Sep 21;11:18696. doi: 10.1038/s41598-021-97022-y (PMC8455665; doi:10.1038/s41598-021-97022-y)
Supplement: Supplementary file 1 — Supplementary Information. [file 41598_2021_97022_MOESM1_ESM.docx]

**Origin of Pyrite Nodules at the Top of the Nantuo Diamictites, Southern China**

Changjie Liu^1,2,*^ and Ying Ling^3^

^1^ Department of Geology & Geophysics, Louisiana State University, LA, USA

^2^ Department of Geosciences, Texas Tech University, TX, USA

^3^ Department of Earth Sciences, University of California at Riverside, CA, USA

*corresponding author: Changjie Liu (Changjie.liu@ttu.edu)


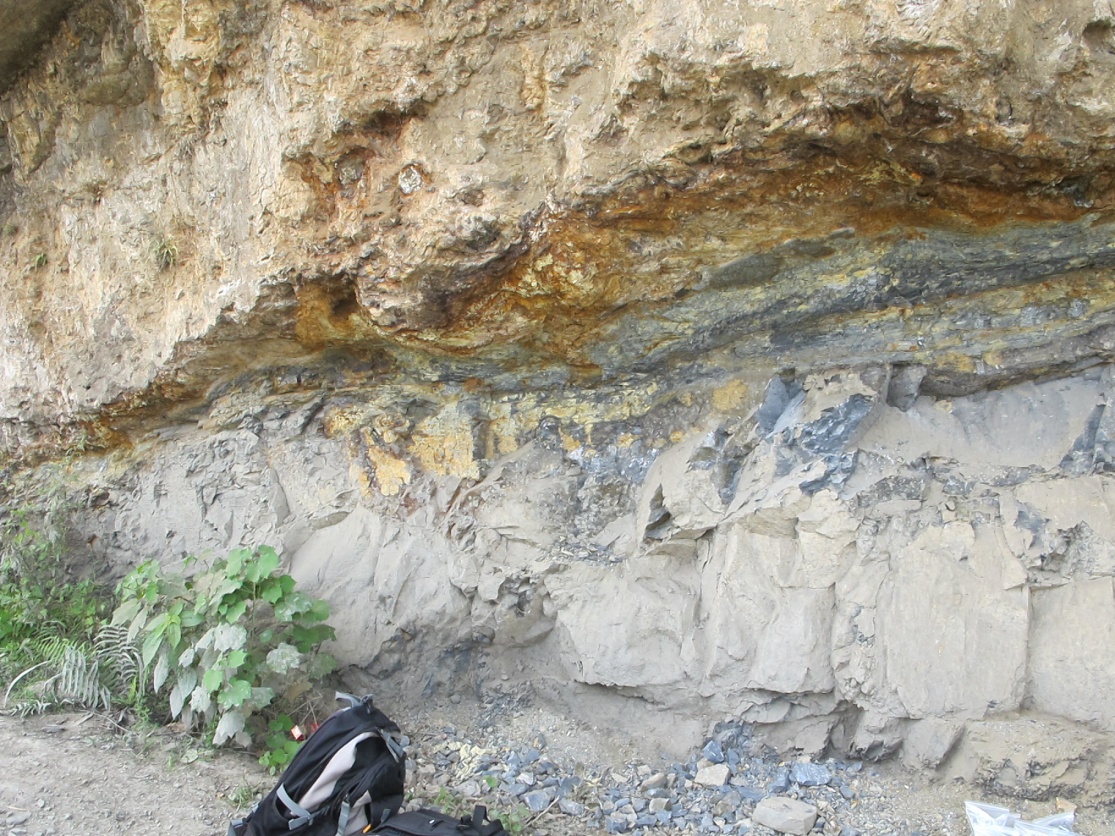

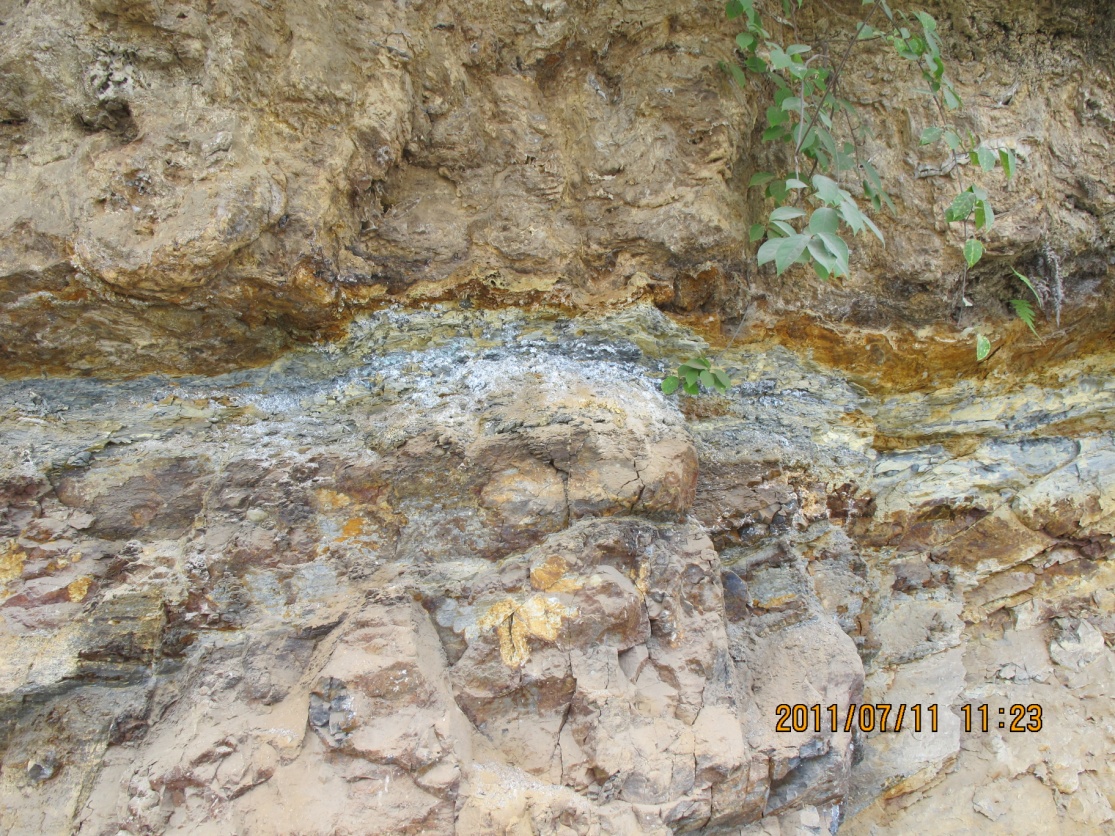


A

B


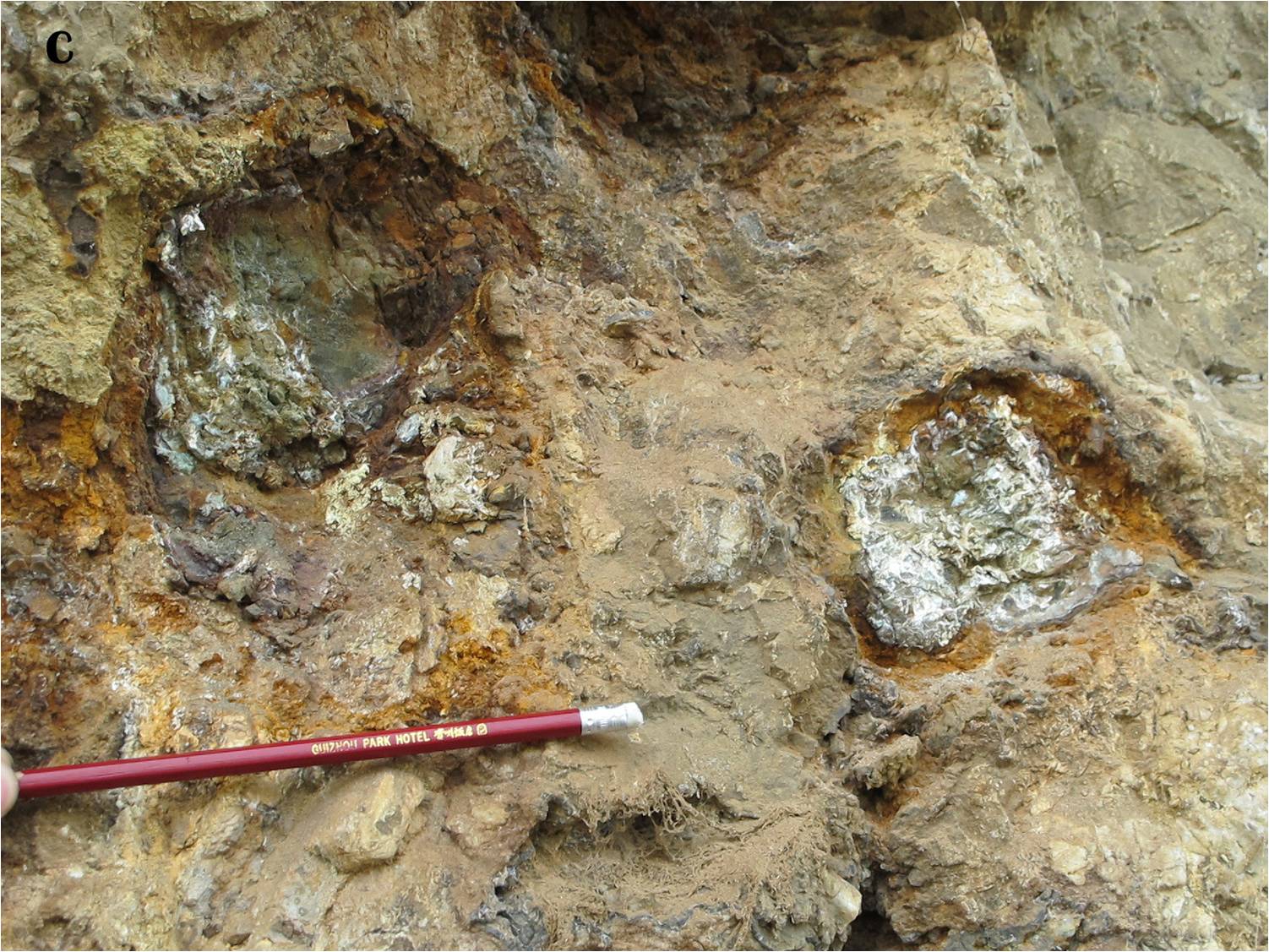

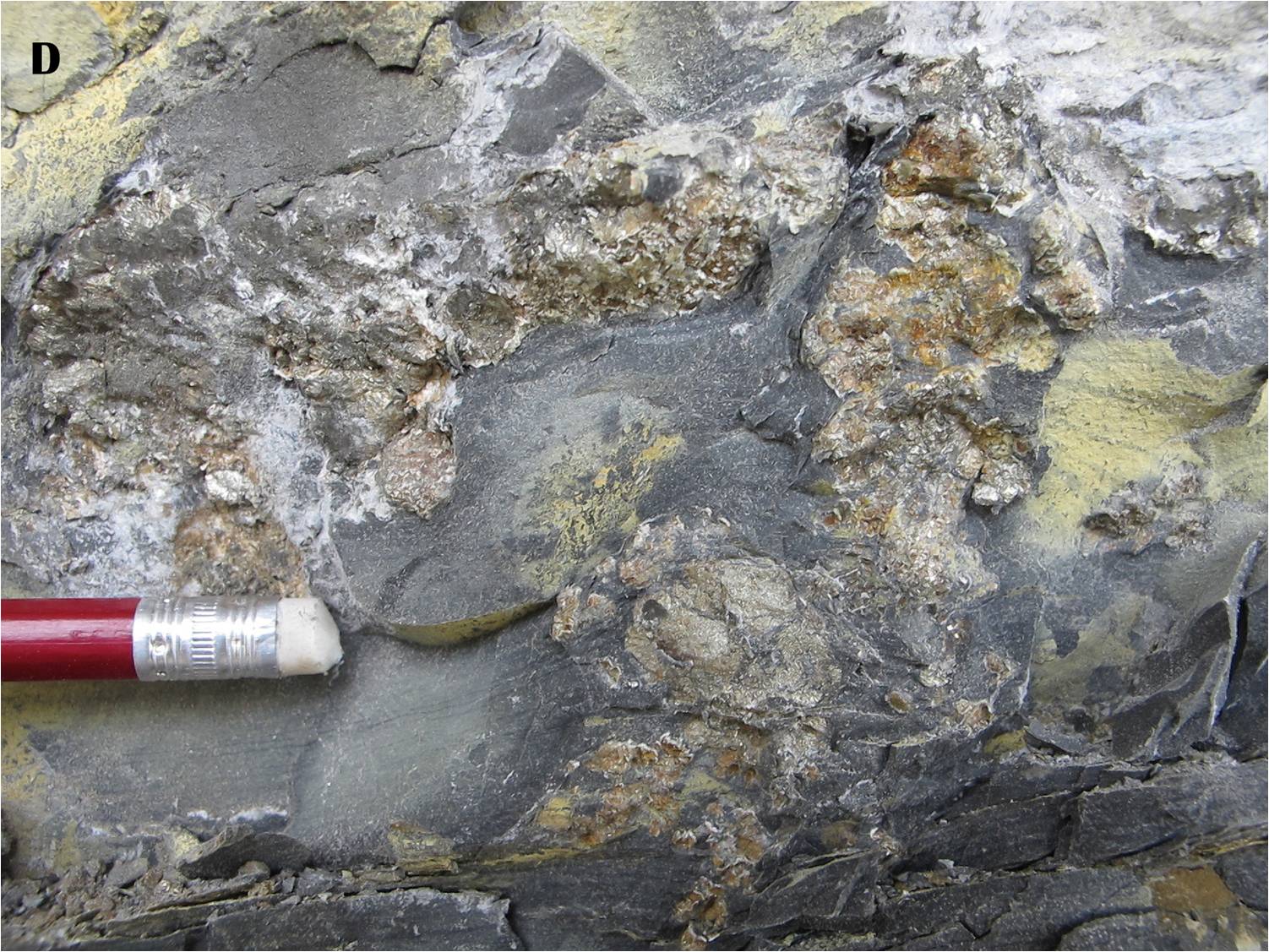


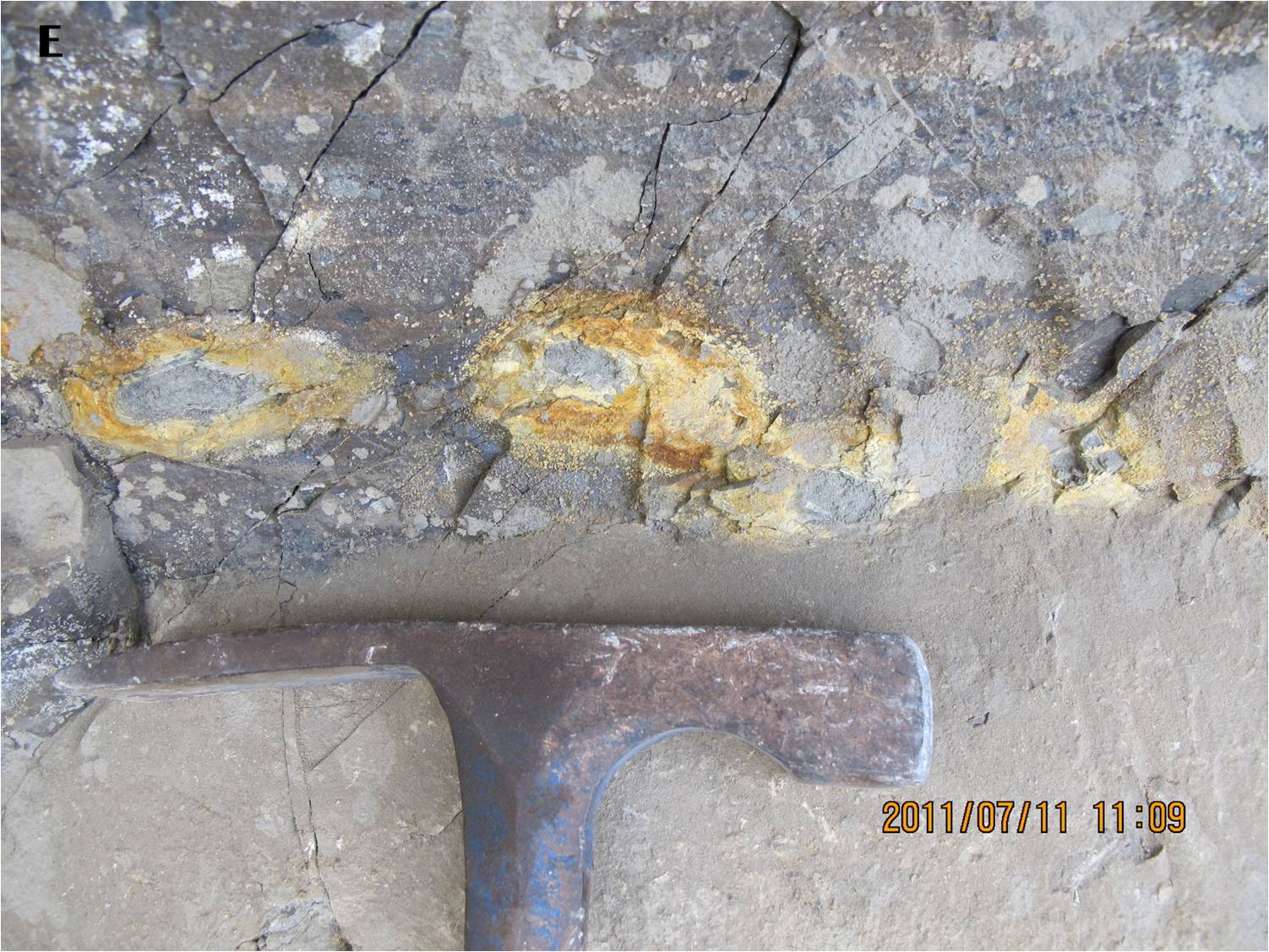

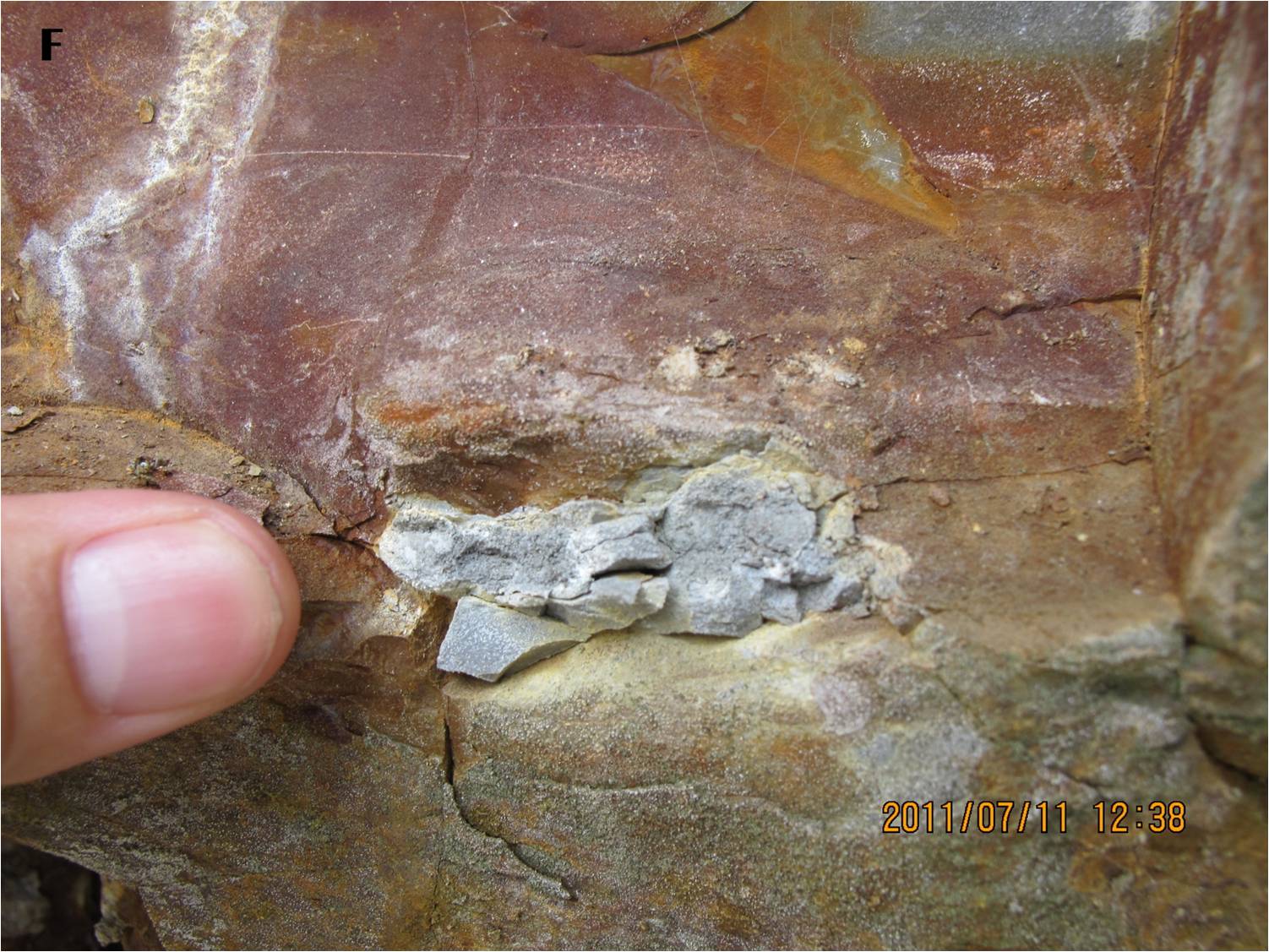


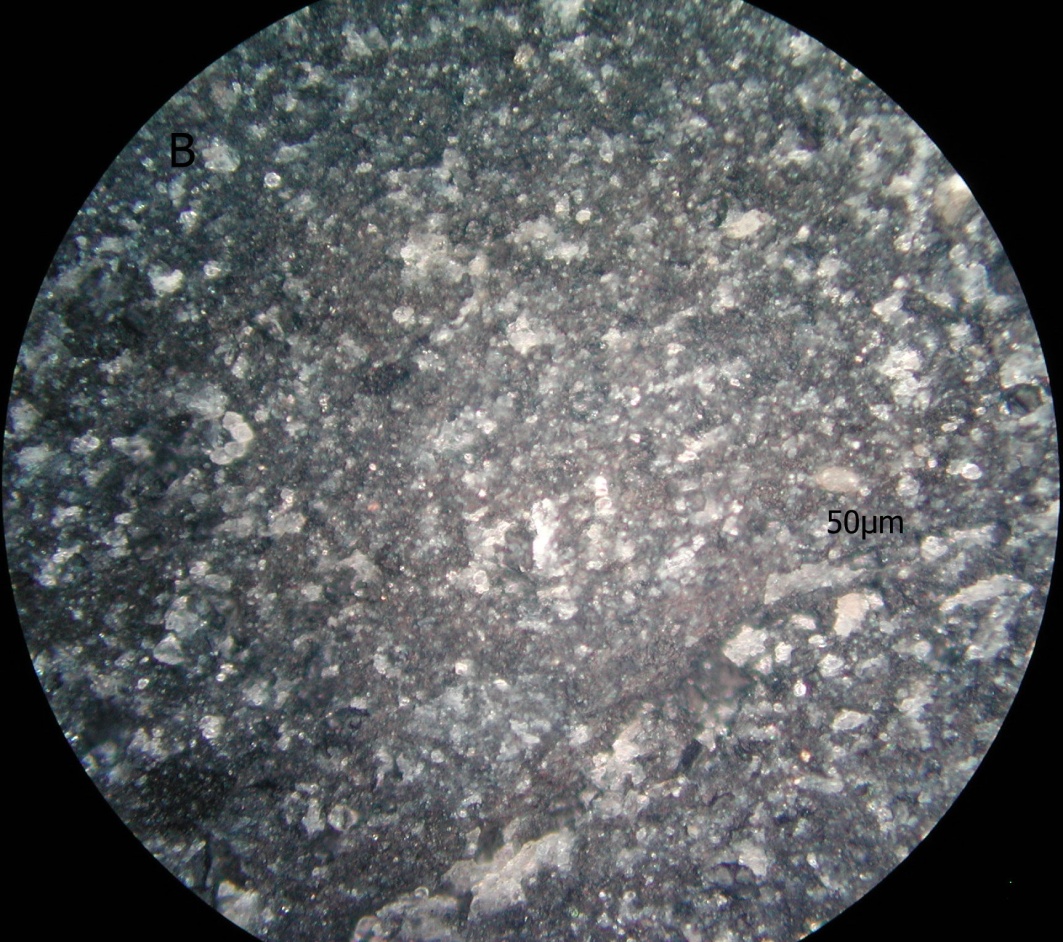

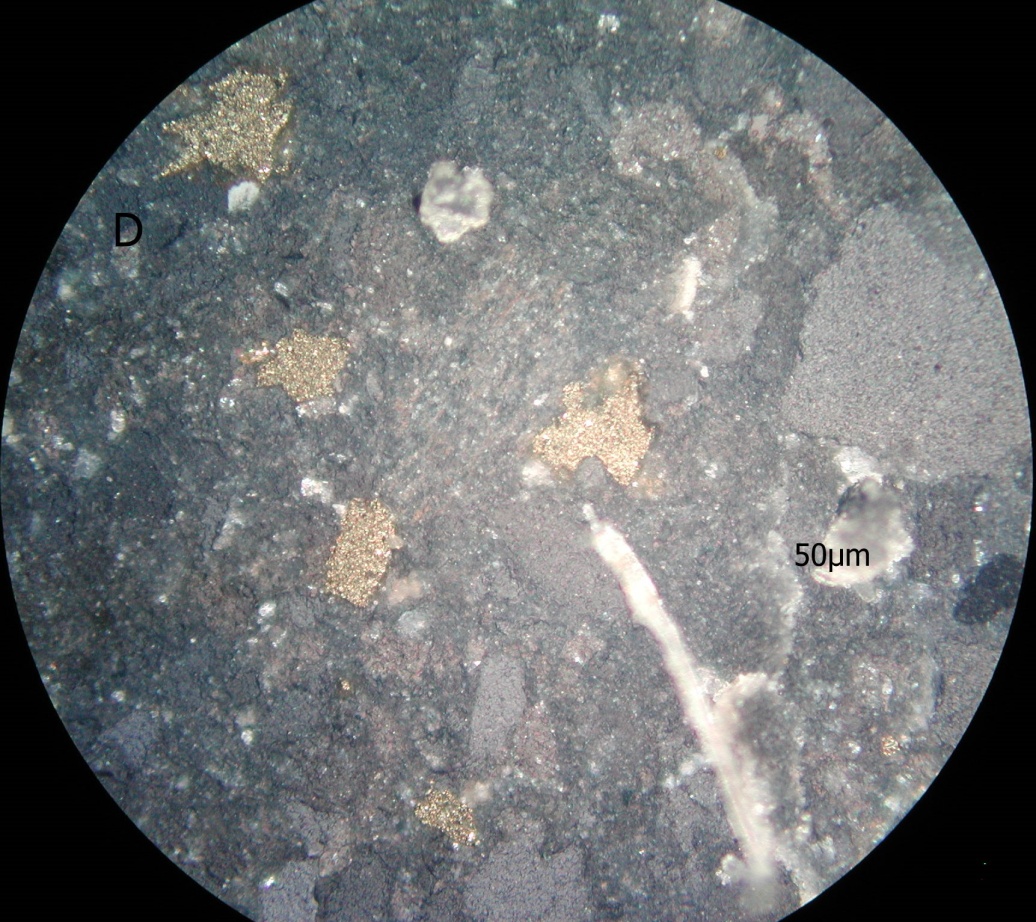
Fig. S1. Field photos of pyrite nodules in Taoying, Guizhou, southern China. A&B: section with pyrite nodules. C: pyrite nodules in the cap dolostones overlying the diamictite. D: irregular pyrite nodules near the top of the diamictite. E: pyrite nodules within the diamictite. F: one pyrite nodule within the diamictite. The width of the pencil and the length of the hammer in the pictures are 0.8 cm and 17 cm, respectively.

pyrite

ZB11-8

ZB11-7


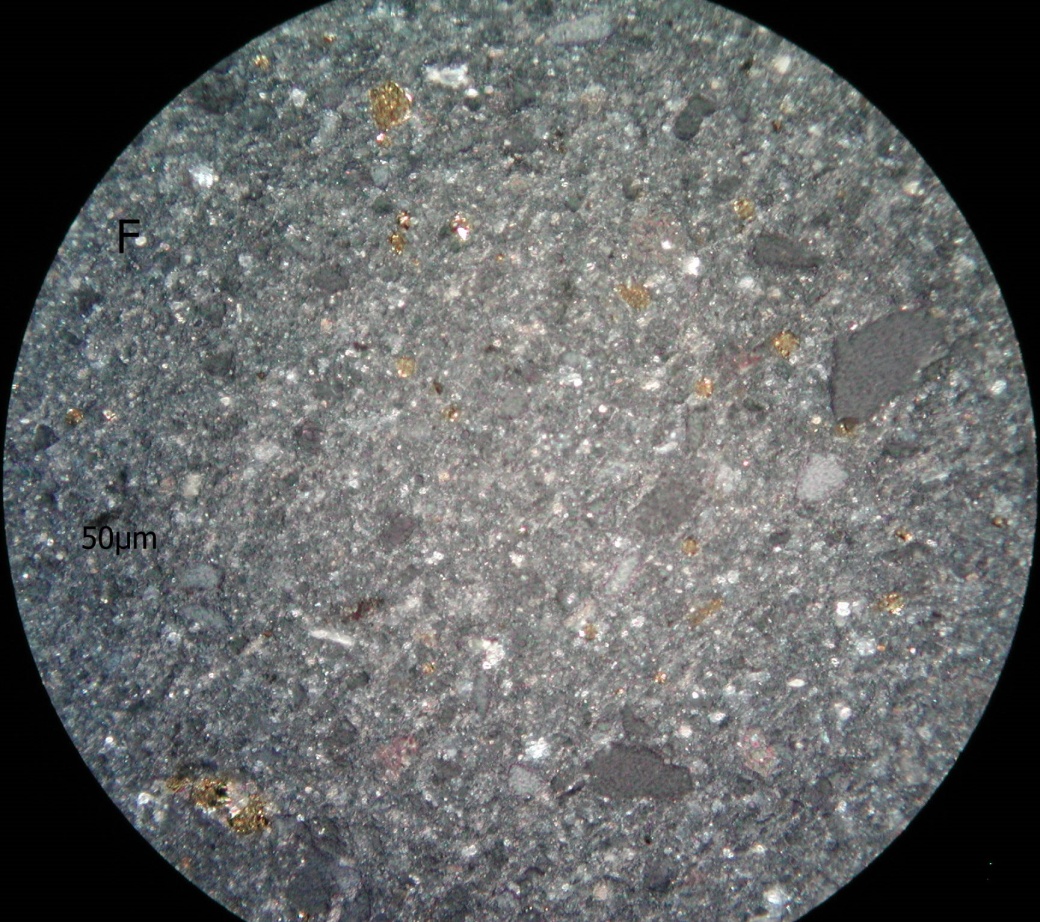


pyrite

ZB11-9


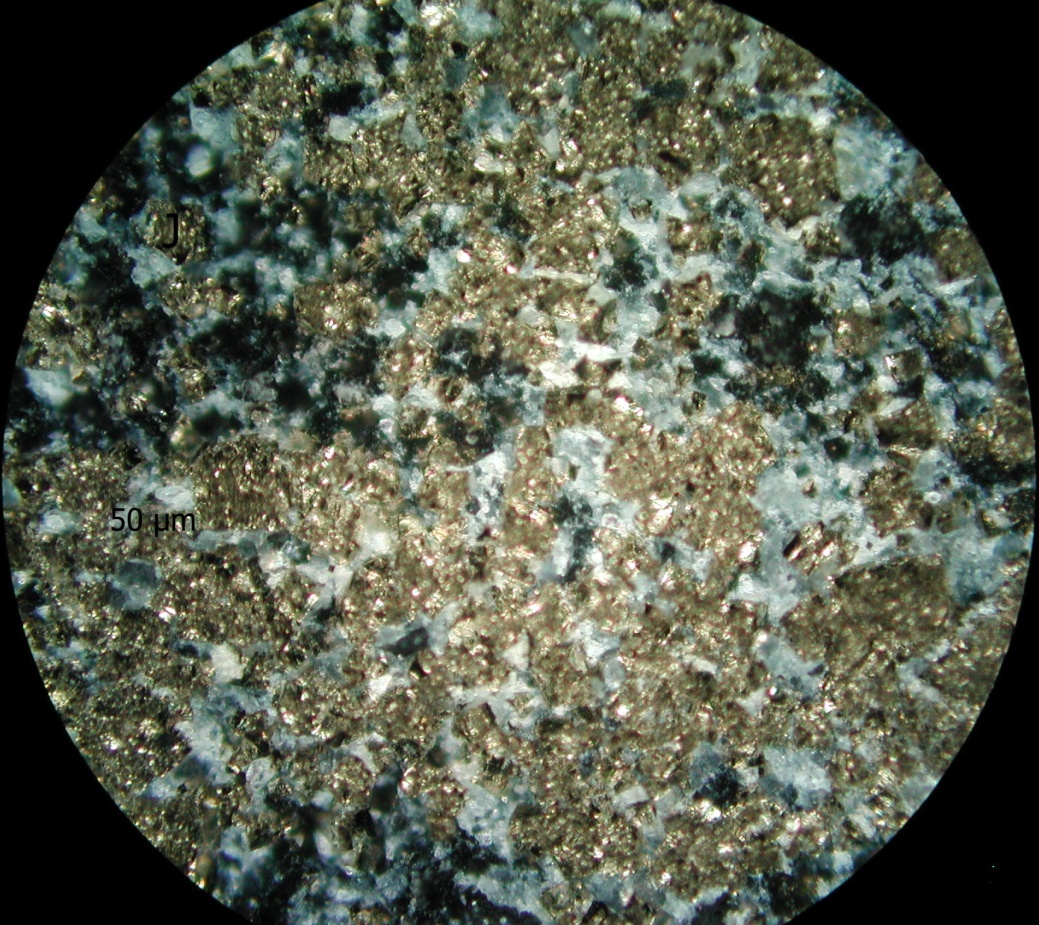


pyrite

ZB11-12


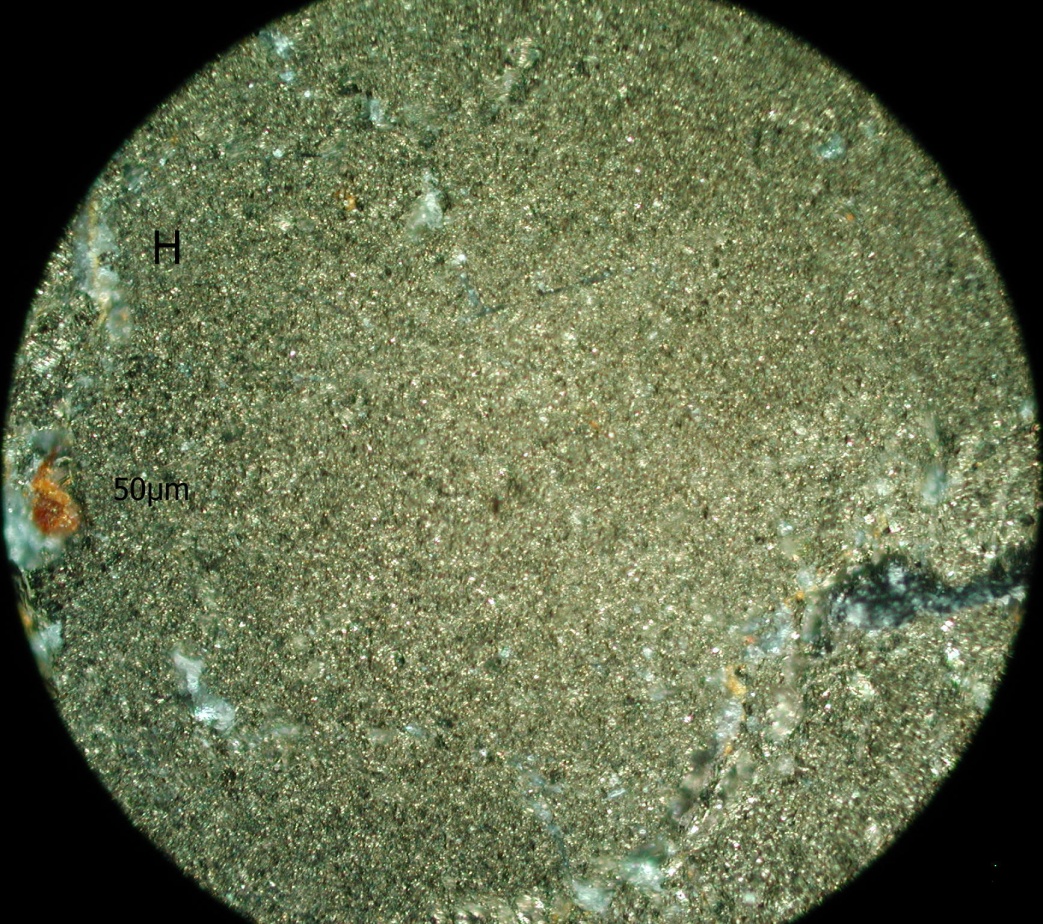


pyrite

ZB11-11


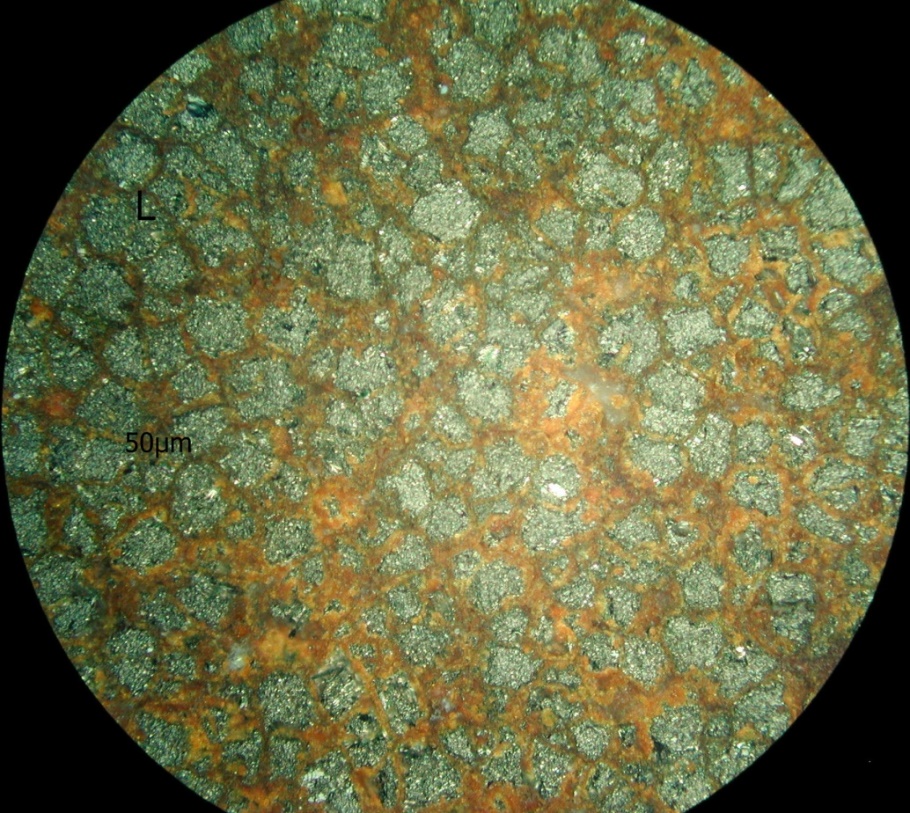


pyrite

ZB11-14

gffff

Fig. S2. Photomicrographs (reflected light) for thin sections of pyrite nodule and bulk diamictite samples. Samples ZB11-7, ZB11-8, and ZB11-9 were bulk diamictite samples. Samples ZB11-10, ZB11-11, and ZB11-12 were pyrite nodules in the diamictite. ZB11-14 was a pyrite nodule in the cap dolostones.











Fig. S3. SEM photos for pyrite nodule samples. Samples ZB11-11 and ZB11-12 were pyrite nodules in the diamictite. ZB11-14 was a pyrite nodule in the cap dolostones.

Fig. S4. Average sulfur isotope values of pyrite nodules at 0.3-1 cm scale (a) and pyrite content in pyrite nodules and diamictite samples (b) at different depth from the top of cap dolostone.


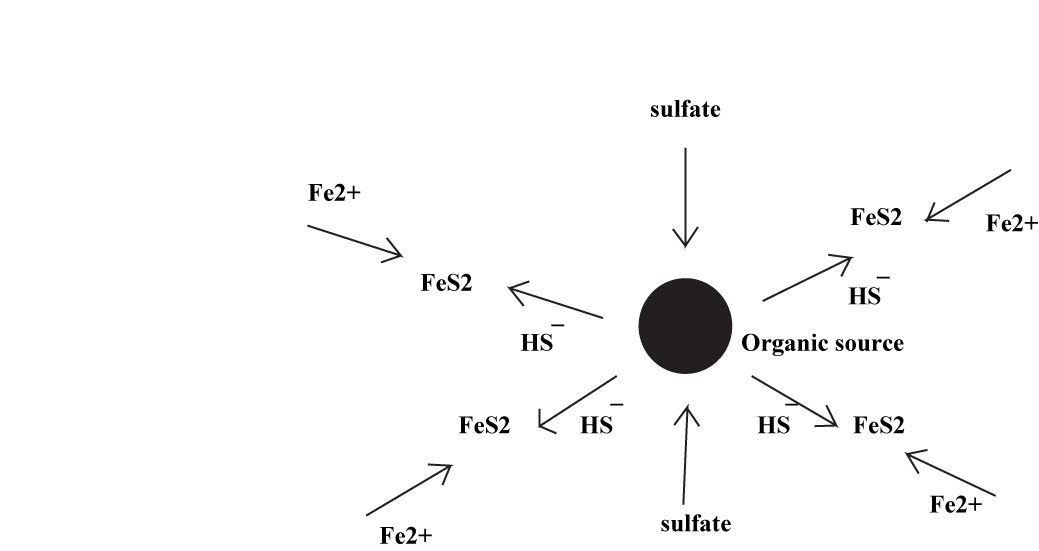


Fig. S5. Scheme showing the pyrite concretion formation^1^. If microbial sulfate reduction takes place in a closed system, the δ^34^S values of the produced sulfide will increase with increasing δ^34^S value of the left-over sulfate^2^.


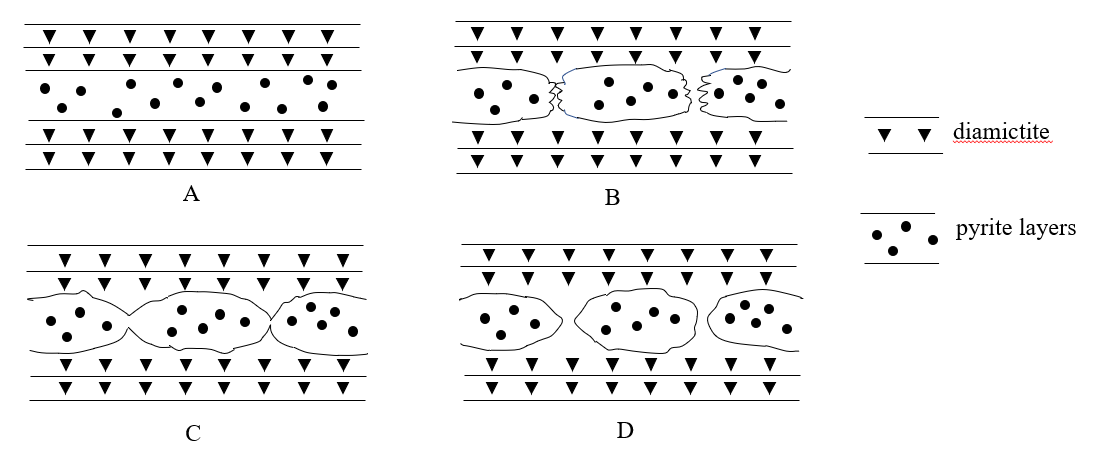


Fig. S6. Scheme showing a possible mechanism of pyrite nodule formation in diamictite: A) pyrite layers are deposited in diamictite; B) pyrite layers pull apart due to differential cementation; C) pyrite layers turn to semi-linked nodules due to sedimentary pressure; D) pyrite nodules are formed.

**References**

1. Berner, R.A., 1969, Migration of iron and sulfur within anaerobic sediments during early diagenesis: American Journal of Science, v. 267, p. 19-42.

2. Kaplan, I.R., and Rittenberg, S.C., 1964, Microbiological fractionation of sulphur isotopes: Journal of General Microbiology, v. 34, p. 195-212
